# Editorial: Endoscopic vacuum therapy (EVT) in the gastrointestinal tract—a paradigm shift in the surgical treatment and prevention of anastomotic insufficiencies and perforations

**DOI:** 10.3389/fsurg.2023.1222993

**Published:** 2023-06-13

**Authors:** Markus M. Heiss, Clemens Schafmayer, Christian A. Gutschow

**Affiliations:** ^1^ Department of Abdominal, Tumor, Transplant and Vascular Surgery, Cologne-Merheim Medical Center, Witten/Herdecke University, Cologne, Germany; ^2^ General, Visceral, Thorax, Vascular and Transplantation Surgery, Rostock University Medical Center, Rostock, Germany; ^3^ Department of Visceral and Transplant Surgery, University Hospital Zurich, Zurich, Switzerland

**Keywords:** endoscopic vacuum therapy, EVT, gastrointestinal wall defect, anastomotic leakage, VACStent, preemptive therapy of anastomotic suture line

**Editorial on the Research Topic** Endoscopic vacuum therapy (EVT) in the gastrointestinal tract—a paradigm shift in the surgical treatment and prevention of anastomotic insufficiencies and perforations

Endoluminal vacuum therapy (EVT) has proven to be a very effective and safe method of treating perforations and suture leaks in both the upper and lower gastrointestinal tract and has gained wide clinical acceptance in recent years (1).

In dehiscences with larger extraluminal wound cavities, intracavitary sponge placement has been recommended to achieve optimal debridement of the contaminated area. However, mediastinal vascular erosion with subsequent hemorrhage or airway fistulas remain a major concern, especially when high negative pressure is used. Therefore, luminal sponge placement, or even conversion to other modalities such as stent or VACStent placement is recommended once the extraluminal cavity is contained and granulated.

However, EVT with luminal sponge placement raises the issue of obstruction, which can be overcome, albeit with difficulty, by placing a feeding tube. The VACStent offers an alternative way to ensure safe closure of the intestinal wall defect while preserving intestinal passage and allowing early enteral nutrition (Schäfer et al.  “*Don't be afraid of black holes: Vacuum sponge and vacuum stent treatment of leaks in the upper GI tract—a case series and mini-review*”). In addition, experience to date shows that the VACStent is easy and safe to use, thus combining EVT with the advantages of covered stenting (Lange et al. “*Clinical implantation of 92 VACStents in the upper gastrointestinal tract of 50 patients—applicability and safety analysis of an innovative endoscopic concept”*). The sponge cylinder around the stent fixes the device to the intestinal wall like a suction cup and seals it against the intestinal lumen together with the somewhat protruding stent ends. In the lower GI tract, the VACStent allows effective EVT while maintaining stool passage and avoiding a stoma.

Recent clinical results show that EVT is indeed able to deliver on this promise and avert the potentially catastrophic consequences of esophageal perforation or anastomotic leakage with success rates of 80-100% (Pattynama et al. “*Vacuum-stent: A combination of endoscopic vacuum therapy and an intraluminal stent for treatment of esophageal transmural defects*”). This is also true for duodenal lesions if the defect is technically and endoscopically accessible (Wichmann et al. “*Endoscopic negative pressure therapy for duodenal leaks”*).

The treatment of gastrointestinal leaks with EVT has reduced both morbidity and mortality, leading to a real paradigm shift. The traditional principle of aggressive surgical therapy plus drainage can now very often be replaced by an endoscopic approach, avoiding extensive, complicated revision surgery with often serious consequences for the patient. EVT is therefore one of the most important innovations in surgery in recent decades.

Nevertheless, the development of EVT is only at its beginning. There is growing evidence that early prophylactic use of EVT can prevent anastomotic leakage or their sequelae in both the upper and lower GI tracts (Schiffmann et al. “*SEVTAR—A multicenter randomized controlled trial to investigate the impact of prophylactic endoluminal placed vacuum sponge for prevention of anastomotic leakage after low rectal resections*”), which can be explained by improved wound healing due to the mechanisms of EVT, especially enhanced microperfusion (2). Even if suture leakage occurs under preemptive EVT, the suction drainage of the sponge or sponge cylinder effectively prevents the formation of a larger wound cavity and the development of sepsis. Anastomotic leakage thus loses its clinical horror and can heal without consequences (Lange et al. “*Preemptive endoluminal vacuum therapy with the VACStent—A pilot study to reduce anastomotic leakage after Ivor Lewis hybrid esophagectomy*”). If this is confirmed in the ongoing and planned studies, EVT would become an integral part of any major surgery which includes anastomosis in endoscopically accessible regions. This would have a significant positive impact on the clinical outcome and the financial burden of surgical complication treatment.
